# Talus insufficiency fracture due to long-term methotrexate therapy in a patient with rheumatoid arthritis

**DOI:** 10.1093/jscr/rjaf780

**Published:** 2025-10-02

**Authors:** Julian Ramin Andresen, Sebastian Radmer

**Affiliations:** Division of Trauma Surgery, Department of Orthopaedics and Trauma Surgery, Medical University of Vienna, Vienna, Austria; Specialist Practice for Orthopedics, Centre for Orthopaedics, Berlin, Germany

**Keywords:** insufficiency fracture, methotrexate, MTX osteopathy, osteoanabolic therapy, rheumatoid arthritis

## Abstract

Insufficiency fractures are a well-known complication in patients with inflammatory rheumatic diseases, but can also be caused by long-term methotrexate (MTX) therapy. We report on a 72-year-old female patient with rheumatoid arthritis and more than 10 years of MTX treatment who was diagnosed with an insufficiency fracture of the trochlea tali without previous trauma. After discontinuation of MTX therapy and initiation of osteoanabolic treatment, there was a distinct clinical improvement with prompt fracture healing. The case underlines the importance of early MRI diagnosis and treatment adjustment in cases of suspected MTX osteopathy to avoid missing or protracted fracture healing and to minimize the risk of further fractures.

## Introduction

Insufficiency fractures in patients with rheumatic diseases are not uncommon, they can be induced both by the chronic inflammatory disease itself and by its drug therapy [1]. In addition to glucocorticoids, which reduce bone density and increase fracture risk even with short-term administration [2], so-called basic therapeutics (DMARDs: disease-modifying antirheumatic drugs) such as leflunomide and methotrexate also have an influence on bone metabolism [3], particularly with long-term treatment of inflammatory rheumatic diseases. While glucocorticoid-induced fractures preferentially occur in the axial skeleton [4], fractures associated with DMARDs tend to occur in the distal lower leg and the tarsal and metatarsal bones [5].

## Case report

We report on a 72-year-old female patient with many years of methotrexate (MTX) therapy due to rheumatoid arthritis, in whom an insufficiency fracture occurred in the trochlea tali.

### Clinical course

**Figure 1:**
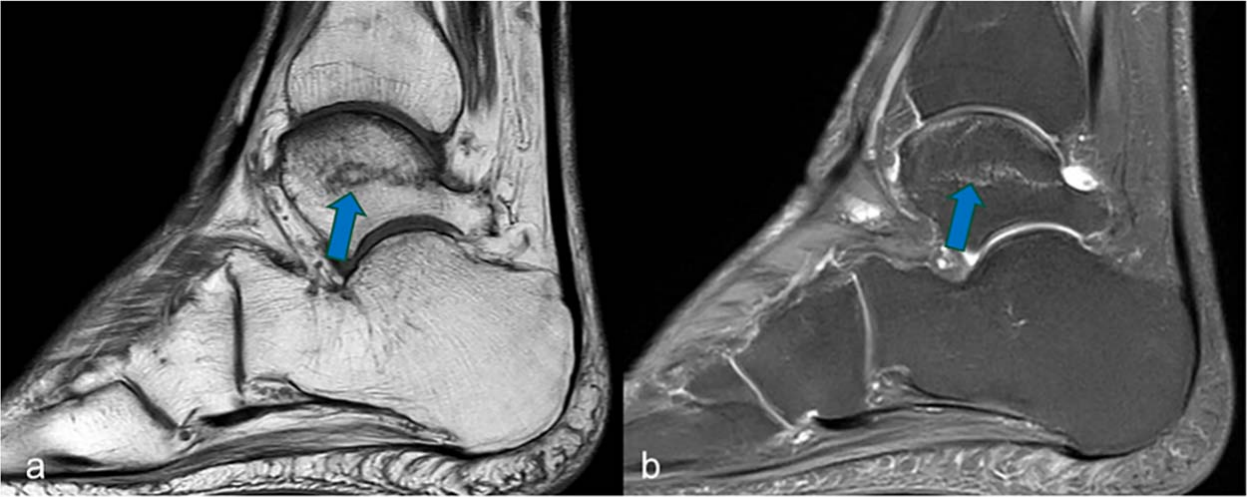
Sagittal MRI cross-sectional imaging showing (a) a T1-weighted and (b) a proton-weighted fat-suppressed image of the right tarsal bones. The ligamentous fracture in the trochlea tali was marked with arrows.

A history of trauma could be excluded. At a dose of 10 mg/week, the duration of MTX therapy was 11 years. An MRI scan performed due to severe pain of 7 on the VAS showed an edema zone with a demarcation line in the area of the right trochlea tali (Fig. 1). A DXA scan (Lunar Prodigy®) showed significantly reduced T-score values of −3.2 for the lumbar spine and −2.8 for the hips. In the area of the thoracolumbar junction, two consolidated sintering fractures were found in the conventional X-ray of the axial skeleton. At the time of the talus fracture, the patient was already undergoing antiresorptive osteoporosis therapy with a bisphosphonate. At 52 nmol/l, the vitamin D level was in the lower normal range at this time.

The patient was immobilized in a short walker and mobilized on crutches with rolling weight-bearing for 6 weeks, after which the walker was removed with an adjusted increase in weight-bearing up to full weight-bearing. MTX therapy was discontinued, and osteoanabolic therapy with Romosozumab (Evenity®) was also administered. After 10 weeks, the pat. was mobilized under full weight-bearing, the pain at this time was VAS 2. The MRI examination revealed individual residual edema of the previously described lesion, new fractures could be excluded.

## Discussion

MTX osteopathy is extremely rare, with fewer than 250 cases currently published worldwide. A case of MTX-induced osteopathy in rheumatology was first described in 1983 in a 72-year-old man who was treated for extensive skin psoriasis and psoriatic arthritis. He spontaneously developed a fatigue fracture of the medial femoral condyle on the right [6]. However, in patients with rheumatoid arthritis who are receiving long-term MTX therapy, MTX osteopathy should be considered if exercise-induced pain and insufficiency fractures of the lower extremity occur [7]. To confirm the diagnosis promptly, an MRI scan of the affected region should be performed, which often reveals typical edema zones and pathognomonic, ligamentous, or meandering fractures [7–9]. In these cases, it is necessary to discontinue MTX therapy to ensure successful treatment [1, 10, 11], which also led to a reduction in pain and healing of the fracture in our patient. In patients with insufficiency fractures due to MTX osteopathy, the continuation of MTX treatment is associated with a high risk of further fractures [11].

The possibility of additional osteoanabolic therapy should be investigated to accelerate fracture healing, improve osteopenic bone texture, and minimize fracture risk [10].

## Conclusion

The present case illustrates the need to consider MTX-induced osteopathy in the differential diagnosis of persistent pain and insufficiency-type fractures of the lower extremity under long-term methotrexate therapy. Early imaging, discontinuation of the triggering medication and, if necessary, osteoanabolic therapy can make a decisive contribution to fracture healing, significantly reduce the risk of further insufficiency fractures and considerably improve the patient's quality of life.

## Data Availability

Data generated during and/or analyzed during the current study are available from the corresponding author on reasonable request.
